# Correction of Retinal Nerve Fiber Layer Thickness Measurement on Spectral-Domain Optical Coherence Tomographic Images Using U-net Architecture

**DOI:** 10.18502/jovr.v18i1.12724

**Published:** 2023-02-21

**Authors:** Ghazale Razaghi, Masoud Aghsaei Fard, Marjaneh Hejazi

**Affiliations:** ^1^Medical Physics and Biomedical Engineering Department, School of Medicine, Tehran University of Medical Sciences, Tehran, Iran; ^2^Department of Ophthalmology, Farabi Eye Hospital, Tehran University of Medical Sciences, Tehran, Iran; ^3^Research Center for Molecular and Cellular Imaging, Bio-Optical Imaging Group, Tehran University of Medical Sciences, Tehran, Iran

**Keywords:** Deep Learning, Optical Coherence Tomography, Retinal Nerve Fiber Layer

## Abstract

**Purpose:**

In this study, an algorithm based on deep learning was presented to reduce the retinal nerve fiber layer (RNFL) segmentation errors in spectral domain optical coherence tomography (SD-OCT) scans using ophthalmologists' manual segmentation as a reference standard.

**Methods:**

In this study, we developed an image segmentation network based on deep learning to automatically identify the RNFL thickness from B-scans obtained with SD-OCT. The scans were collected from Farabi Eye Hospital (500 B-scans were used for training, while 50 were used for testing). To remove the speckle noise from the images, preprocessing was applied before training, and postprocessing was performed to fill any discontinuities that might exist. Afterward, output masks were analyzed for their average thickness. Finally, the calculation of mean absolute error between predicted and ground truth RNFL thickness was performed.

**Results:**

Based on the testing database, SD-OCT segmentation had an average dice similarity coefficient of 0.91, and thickness estimation had a mean absolute error of 2.23 ± 2.1 μm. As compared to conventional OCT software algorithms, deep learning predictions were better correlated with the best available estimate during the test period (r^2^ = 0.99 vs r^2^ = 0.88, respectively; *P* < 0.001).

**Conclusion:**

Our experimental results demonstrate effective and precise segmentation of the RNFL layer with the coefficient of 0.91 and reliable thickness prediction with MAE 2.23 ± 2.1 μm in SD-OCT B-scans. Performance is comparable with human annotation of the RNFL layer and other algorithms according to the correlation coefficient of 0.99 and 0.88, respectively, while artifacts and errors are evident.

##  INTRODUCTION

Treatment of retinal diseases can be greatly improved by early diagnosis and monitoring of optic neuropathies. Glaucoma and other optic neuropathies can be diagnosed based on assessing the thickness of the retinal nerve fiber layer (RNFL). ${}^{\left[1--3\right]}$ RNFL thickness can now be measured quantitatively with optical coherence tomography (OCT) software which is a convoluted imaging technology.

RFNL thickness is currently measured automatically by spectral domain OCT (SD-OCT) using segmentation algorithms. However, despite improvements in SD-OCT hardware and software, RNFL segmentation errors are still rather common. According to the literature, artifacts or segmentation errors can be found anywhere from 19.9% to 46.3% of SD-OCT scans of the RNFL.^[4,5]^There are several factors associated with these segmentation errors in OCT images, which include image decentration, epiretinal membranes, long axial lengths, and poor visual acuity.^[5]^ Although manually correcting the segmentation errors is possible, accomplishing this in a busy clinical practice could be infeasible due to the lengthy time commitment.^[6]^

SD-OCT has been applied in the diagnosis and segmentation of RNFL throughout several recent studies that used deep learning (DL) models.^[7]^Devalla et al developed a DL technique that allowed for more precise optic nerve head tissue segmentation than the manual method.^[8]^ A higher level of accuracy (ACC) was achieved by the algorithm as compared to manual segmentation performed by two graders. Accordingly, this algorithm yielded 8.85 ± 3.40% and 9.01 ± 4.20% errors for RNFL thicknesses calculations, while between the two graders, 5.94 ± 2.30% errors were observed. Thompson et al in their article demonstrated that glaucomatous eyes could be distinguished from healthy eyes by training a DL algorithm on raw SDOCT B-scans.^[9]^ With an area under the receiver operating characteristic (ROC) curve of 0.96, the proposed algorithm is superior to the conventional RNFL thickness parameters used in the instrument's printout (*P* < 0.001).

In another study by Ma et al,^[10]^ U-net with residual block for RNFL thickness estimation was used in raw OCT images. They achieved an acceptable correlation, and the Dice Similarity Index was 0.92 for test samples. In order to quantify the thickness of the retinal nerve fiber layer on OCT images for three test set groups, Mariottoni et al^[11]^ provided a DL segmentation-free method based on ResNet34 that had been pre-trained on the ImageNet dataset. The 2D-OCT scan has been used without a training mask and any previous biomarker definition as input in segmentation-free approaches. Also, Medeiros et al^[12]^ and An et al^[13]^ obtained the average thickness directly without segmentation according to their network design on thickness maps and fundus images. The clinical relevance of examining trends in retinal layer thickness changes and retinal structure deformation is due to the fact that for some diseases, changes in RNFL thickness for a specific period are less than the axial resolution of OCT. This thickness change cannot be determined by OCT software, but DL is able to do so. Therefore, DL-based methods let clinicians explore the disease progression in the early stages. Accordingly, some studies have concentrated on segmenting more than one layer. Fang et al used a hybrid convolutional neural network (CNN) model for segmenting nine retinal layer boundaries in age-related macular degeneration (AMD) patients,^[14]^ and Pekala et al designed CNN in DenseNet architecture for retinal OCT segmentation.^[15]^

To reduce the segmentation error, we used a DL algorithm based on convolution to determine the average RNFL thickness in this study. Our proposed method can be considered as a more robust method of RFNL thickness estimation than the conventional segmentation algorithms as DL segmentation according to our hypothesis, would provide more accurate measurements of RNFL thickness for images which have segmentation errors.

To develop and evaluate a system that is reliable at measuring RNFL layer thickness, this study aims at developing and evaluating a DL system. With enough database of SD-OCT images to prevent overfitting, DL models were trained on OCT images to illustrate the algorithm's ACC and reliability in analyzing and quantifying the thickness of the RNFL.

##  METHODS

This study used DL to develop an algorithm for measuring RNFL thickness. Figure 1 summarizes our proposed workflow. The first step involved reducing speckle noise using a preprocessing method. CNNs were then used to delineate RNFL contours. A morphological method was applied to remove any inappropriate patterns in segmentation results. Finally, RNFL thickness was determined according to our DL rules.

**Figure 1:**
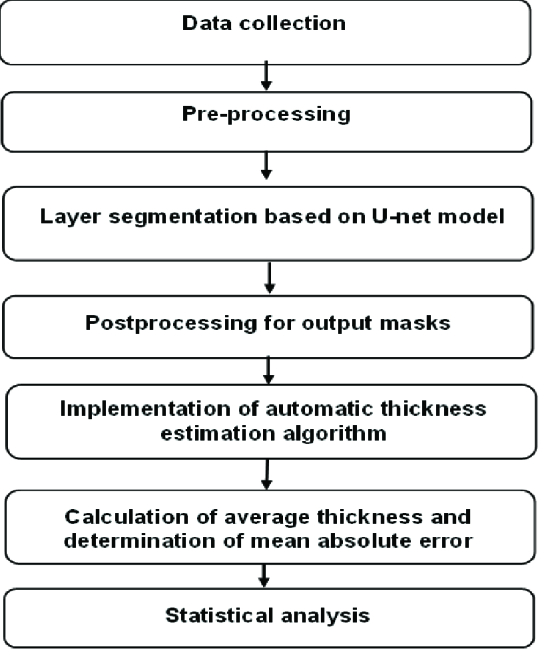
Flowchart for steps in material and methods.

### Data Collection 

From 90 left eyes and 62 right eyes of participants (age range of 20–80 years), SD-OCT images of healthy and unhealthy (glaucomatous optic neuropathy) patients were collected. The Heidelberg OCT machine at the Farabi Eye Hospital captured all of the data, which was anonymized to fulfill the Human Ethics criteria of the Tehran University of Medical Sciences. All 500 images in the dataset were randomly divided into training (80%, 400 OCT images) and validation (20%, 100 OCT images) groups, and 50 OCT images were used in the test group.

### Pre-processing

To prepare OCT images for segmentation, we applied a preprocessing step after extracting each image. Preprocessing was primarily focused on reducing the speckle noise. To minimize these image artifacts, morphological opening filters (square and kernel size 3 × 3) in OpenCV (version 4.5.1, https://opencv.org, Gary Bradsky 1999) were used. The image signal-to-noise ratio factor improved from 25 to 40 dB as a result of this method.

Images must match the input size of a network in order to train it and make predictions on test data. Therefore, images were resized to 256 × 256 pixels with zero padding. Preprocessing becomes more crucial if the dataset contained a limited amount of data. In our study, reducing speckle noise allowed the network to learn useful information like RNFL boundaries efficiently.

### Retinal Nerve Fiber Layer Segmentation 

After preprocessing the image, it is first necessary to segment RNFL accurately to measure the thickness of the fiber layer. An established image segmentation network, U-net, is used to segment RNFL accurately in this study. The U-net created by Ronneberger et al^[16]^ (https://lmb.informatik.uni-freiburg.de/people/ronneber/u-net) has robust performance in the absence of adequate training data. The U-net has advantages in performing segmentation tasks. First, this model allows for simultaneously using global location and context. Second, it performs better for segmentation tasks even with a few training images. Another advantage is that U-net uses a loss function for each image pixel, which helps quickly identify individual cells within the segmentation map.

U-net's detailed network architecture is shown in Figure 2. As input, X is passed to the network, and at the last convolution layer, a binary mask is emitted by the network that includes the RNFL region. The U-net architecture has skip connections to connect encoders and decoders. X's resolution is downsized by a factor of two in the encoder module using Max-pooling for the purpose of capturing contextual details at different resolution steps, and then by up-sampling using Up-Convolution, the resolution is restored in the decoder module, enabling precise localization. Moreover, the architecture shows that the input images are passed through the model and then followed by a series of convolutional layers with the ReLU activation function. In the encoder architecture, we also have multiple convolutional layers with an increasing number of filters (16, 32, 64, 128, 256). We notice that, as we progress toward the decoder, the number of filters in the convolutional layers decreases along with a gradual upsampling of the following layers toward the top. The neural network was carried out with Python programming language (Python 3.5 Software Foundation, https://www.python.org/).

In our U-net network, we used Adam with a default learning rate as an optimizer and binary cross entropy as a loss function. The batch size was equal to 16 with 100 epochs, and we saved the network weights from the “best” epoch by checkpoint and earlystop function.

The training images were manually segmented under the supervision of an expert ophthalmologist using Labelme (http://labelme.csail.mit.edu) to create ground truth masks. With the RNFL OCT images and their respective ground truth masks which were prepared by Labelme, at the last step, the U-net model was trained and validated. We used our trained and validated model to predict the output of the RNFL images in the test set without the corresponding label. Predictions were compared with ground truth masks for analyzing and determining model operation on the test set.

**Figure 2:**
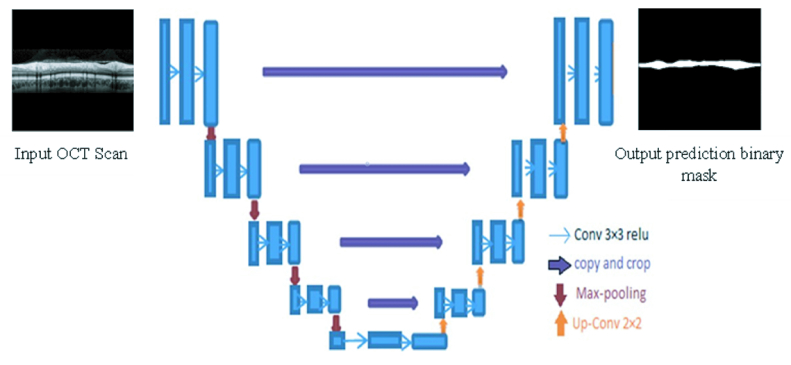
Summary of the U-net architecture. The network receives input X and generates a prediction mask.

### Postprocessing

The generated binary masks may contain gaps and speckles as a consequence of implementing the segmentation algorithm. Morphological algorithms were applied as post-processing methods to fill the gaps. After applying edge detection filters such as the binary threshold or Canny on binary masks to detect white objects from a black background, the "findCountours" function in OpenCV can be used to find continuous contours. It looks for borders and pixels with similar intensities to identify contours.

### Average Thickness Estimation

Following the post-processing phase, the average thickness of the RNFL was determined using the Python environment and the Euclidean distance transform (EDT)^[17,18]^ approach.

A binary digital image was subjected to the EDT to determine the distance between each non-feature (non-zero pixels) and each feature (zero pixels, i.e., RNFL contour). A numerical value is assigned to each binary image pixel by the EDT method indicating how far the black pixel is from the nearest white pixel of the image. For the 2D cases with 256 × 256 pixels, the EDT metric is fast enough to create a distance map for output binary masks.

To find the centerline of the RNFL, we implemented the Skeleton algorithm on EDT outputs. Skeleton is a thinning operation that reduces an object region in EDT output to a matrix of one row. This matrix preserves the significant pixel information (maximum pixel value in EDT results) of the RNFL region.

As stated in the formulas below, the average thickness of RNFL is calculated according to Eq. 2 and as it indicates the mean of maximum values are determined based on Eq. 1. If a ${}_{1}$ , a ${}_{2}$ ,...a ${}_{n}$ is the maximal distance values that were extracted by the skeleton algorithm from the EDT result, to get the thickness diameter, the mean distance value calculated in Eq. 1 was multiplied by two in Eq. 2. In Eq. 1 “*n*” is the total number of maximum values (“*n*” is equal to the number of columns in output mask). In Eq. 2, *F* is the factor that depends on the resolution of the OCT system. In our study, the axial resolution was 2.8 μm. 


$$meanofmaximumvalues=\frac{a_{1}+a_{2}+\cdots +a_{n}}{n}$$

averagethickness=mean×2×F

### Performance Metrics

We used a variety of metrics to measure the OCT segmentation model's performance, including ACC, sensitivity (SEN), specificity (SPE), and dice similarity coefficient (DSC). In the resulting binary mask, SEN, and SPE correspond to the percentages of correctly classified pixels. According to Eq. 3 and Eq. 4, SEN and SPE depend on pixels classification by the number of true positive (TP), false negative (FN), true negative (TN), and false positive (FP) pixels. The significant alert here is that > 50% of the pixels in our output masks are black and are in the background class, so if the U-net model only predicted background correctly, the ACC, SPE, and SEN are more than 0.5, and it can lead to a huge mistake. Since DSC is a combination of SEN and SPE, it stands out more from the other metrics for measuring this task. 


$$SEN=\frac{TP}{TP+FN}$$

$$SPE=\frac{TN}{TN+FP}$$

The DSC is another established metric for comparing binary masks resulting from image segmentation with their ground truth counterparts. The equations of the DSC metric are written as: 


$$DSC=\frac{2\times SEN\times SPE}{SEN+SPE}$$

Figure 3 shows the DSC metric by an example. As shown in Figure 3, the DSC Index was calculated by multiplying the overlap (between the prediction and the ground truth) and dividing it by both areas (of the prediction and the ground truth).

**Figure 3:**
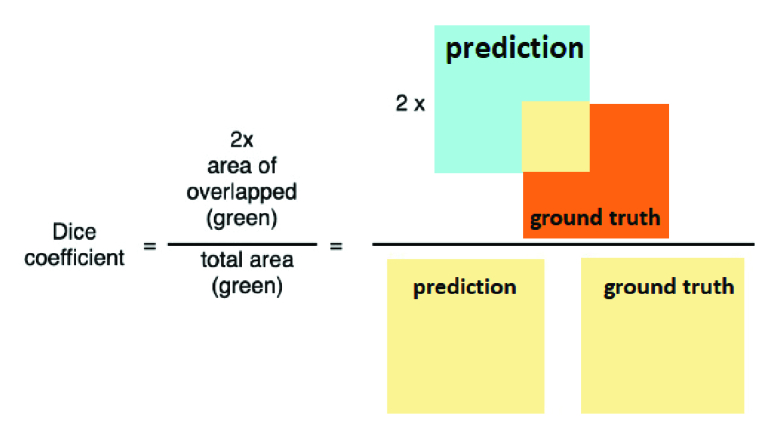
Dice coefficient calculation formula.

##  RESULTS 

To predict the average thickness of the RNFL, we developed a DL algorithm based on U-net and trained using SDOCT B-scans. To compare algorithm results with the best estimate of RNFL average thickness determined by the ophthalmologist, dice coefficients and mean absolute errors (MAE) were calculated.

We have two steps for effective examination, the first step being the DL model evaluation, and the second being consideration of the thickness measurement algorithm performance. Figure 4 shows some examples generated by the proposed methodology on the dataset validation, where it was observed that our U-net model was able to extract the boundary of RNFL at different thicknesses. To prove the validity of the proposed, the test set was used, and Figure 5 shows the segmentation results for two samples in the test set.

To evaluate quantitatively the U-net performance on the validation and the test sets, SEN, SPE, and DSC were utilized as evaluation metrics. Table 1 lists the evaluation results obtained by using the proposed framework on validation and test sets.

Testing and validating images with the U-net model demonstrated a high level of performance. As shown in Table 1, the DSC Index between segmentation results and manual segmentation by an expert was 0.93, and for the test images, the DSC Index was 0.91.

**Figure 4:**
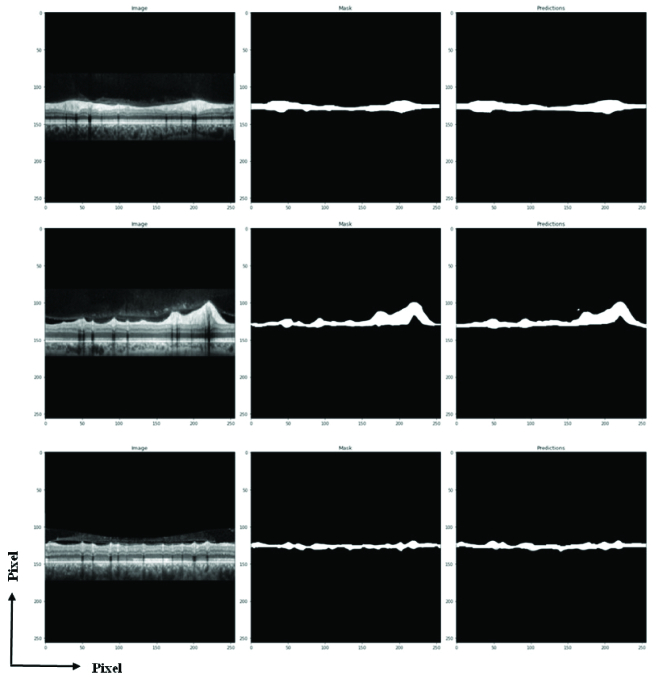
U-net output on validation data. Original image, manually segmented image, and output image, in that order.

In the second step, the average RNFL thickness resulting from the model and thickness determined by the conventional algorithm were compared with the reference RNFL thickness measured by the expert on the test images. A strong correlation was found between the DL segmentation estimates of RNFL thickness and the best measurement of RNFL thickness (Pearson's *r* = 0.996; *P* < 0.001), with an MAE of 2.23 ± 2.1 µm. Figure 6 shows the fluctuation of absolute error for the test images. In addition, the algorithm was not affected by other factors, such as gender or race.

Figure 7 presents a scatter plot between the U-net prediction thickness values and measured thickness values by an expert from 50 SD-OCT B-scans. Based on the test data, a linear regression model is fitted with an R-squared value of 0.9919. As a result, the predicted values are highly linearly related to the measured values, showing that despite the small variance, the predicted thickness values are reliable.

To demonstrate OCT software function, average thicknesses resulting from conventional software were compared with the thicknesses which were estimated by an expert. Figure 8 illustrates the relation between OCT software thickness prediction and the best thickness value recognized by an ophthalmologist.

A linear regression model fitted to the data yields an R-square of 0.8811 and MAE was 9.12 ± 6.9 μm which has a significant error according to RNFL thickness in normal and abnormal OCT images.

**Figure 5:**
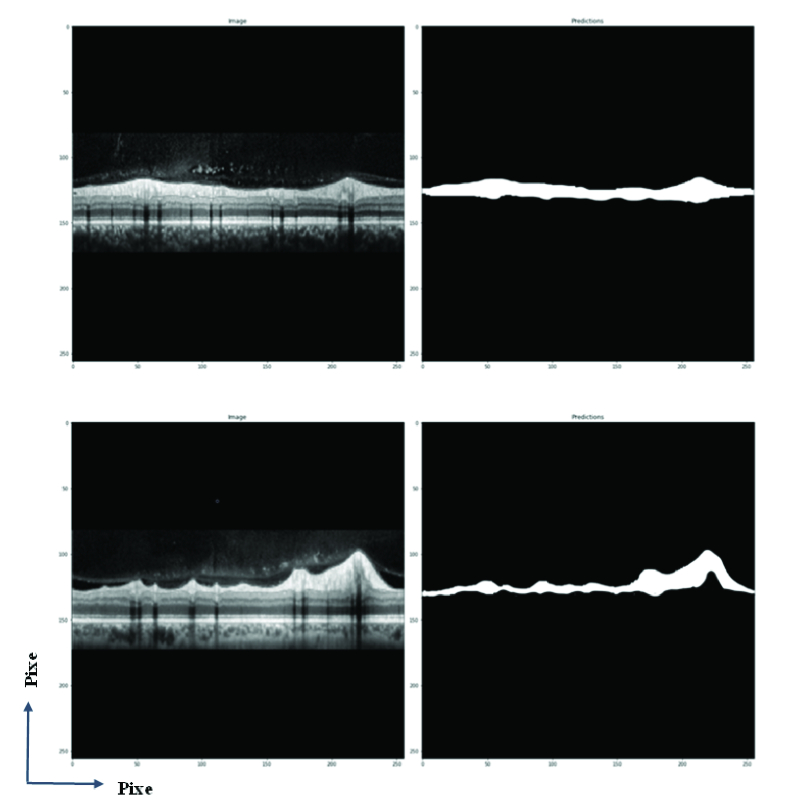
U-net prediction for two samples of the test set. Original image and U-net prediction.

**Table 1:** DSC, SPE, and SEN value for U-net model on validation and test images.


orange**Metric**	orange**SEN**	orange**SPE**	orange**Dice**
Validation	0.94	0.93	0.93
Test	0.93	0.90	0.91
	
	
white<bcol>4</ecol>SEN, sensitivity; SPE, specificity; DSC, dice similarity coefficient

**Figure 6:**
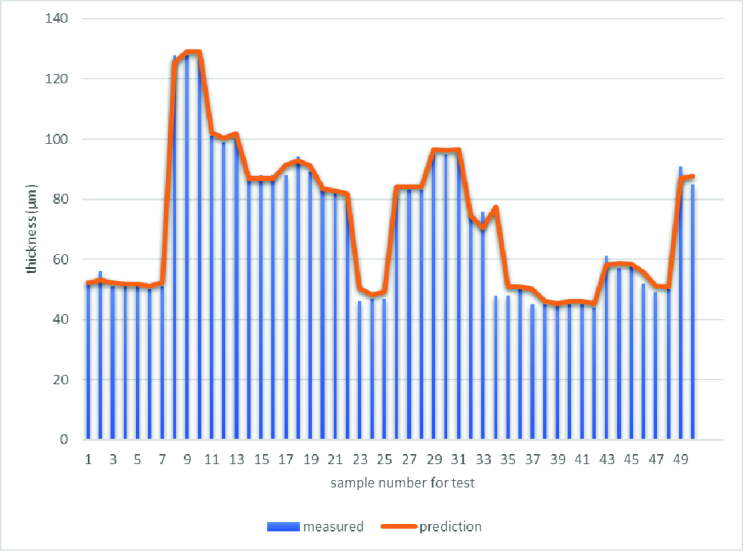
Thickness absolute error between prediction results and best measurement by the expert for each sample in the test set. MAE for 50 samples is equal to 2.23 ± 2.1(μm)..

**Figure 7:**
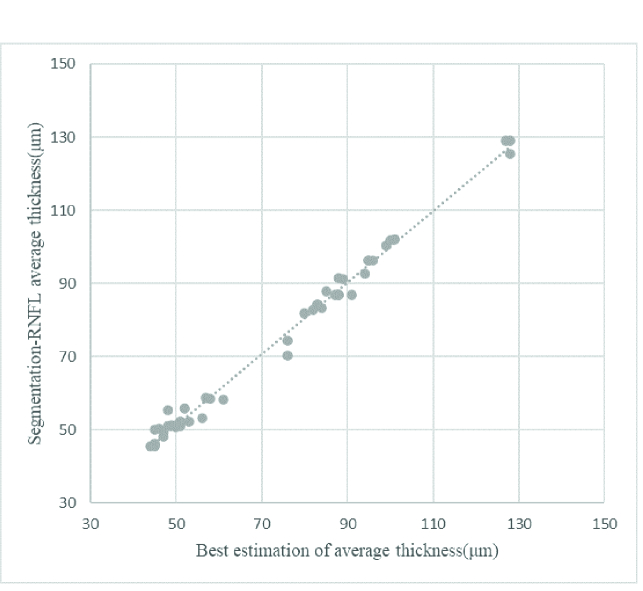
Scatterplot illustrating the relationship between the prediction thickness value and best estimation thickness measurement by the expert for the test set.

**Figure 8:**
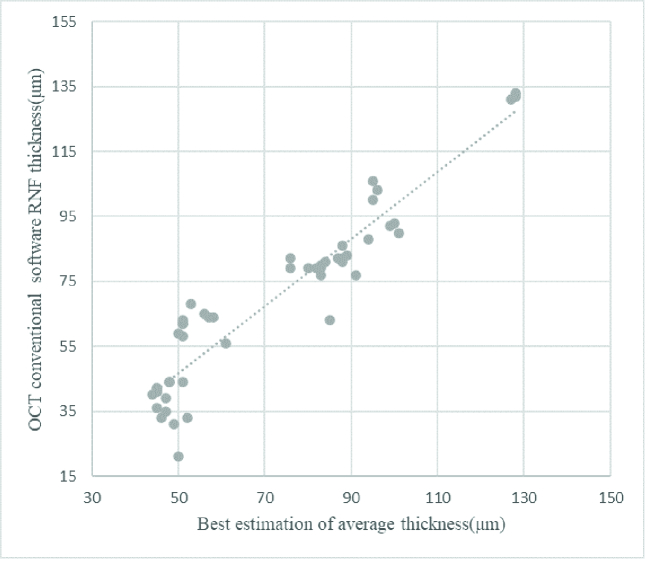
Scatterplot illustrating the relationship between the conventional OCT software and the best estimation thickness measurement by an expert for the test set.

##  DISCUSSION

In the present study, we developed a segmentation DL algorithm capable of predicting RNFL average thickness from B-scans in this study. There was a strong correlation between algorithm estimates of RNFL average thickness and expert measurements of RNFL thickness. On normal images without artifacts, conventional software performed well, but DL-based segmentation estimated RNFL average thickness that is significantly closer to the ground truth values for RNFL thickness than conventional segmentation software. On the test set, the dice coefficient is 0.91, and the MAE is 2.23 ± 2.1 μm in this study.

Several U-net-based models have recently been proposed with promising results for retinal layer segmentation. Devalla et al developed DRUnet for retinal segmentation.^[8]^ The resulting RNFL thickness provided by this algorithm had an error of 8.85 ± 3.40% and at 9.01 ± 4.20% when compared to each grader, while the graders had an error of 5.94 ± 2.30% between each other. Thompson et al found an area under the ROC curve of 0.96 vs 0.87 for the global peripapillary RNFL thickness (*P* < 0.001). Ben-Cohen et al detected four retinal boundaries using a combination of U-net's fully convolutional network, Sobel's edge detection, and graph search.^[19]^ The Dice index in this study for RNFL was 0.95, and the mean difference for thickness was 1.12 pixels. Also, the mean difference for OCT explorer was 3.65 pixels. LF-Unet,^[20]^ U-net++,^[21]^ and ResU-net^[22]^ are other models for segmentation of more than one layer in retinal images; dice scores were 0.83, 0.88, and 0.92, respectively, in comparison to our Dice index that is equal to 0.91. Ma et al proposed U-net with residual blocks and received 0.92 for Dice when adding transfer learning to the model and R^2^ was 0.98 in this study, but we found 0.99 for DSC.^[10]^ Whereas prior SD-OCT segmentation methods based on DL mostly focused on enhancing segmentation performance, our research demonstrates that efficient thickness estimate algorithms are also crucial.

Other studies that work with OCT images received reliable results but U-net model is easier for clinical application because U-net does not have complexity and does not need much space in memory and GPU systems. The response of U-net is fast enough and results are accurate in comparison to conventional software. As you can see in Figure 5, at least 15–20% of images have an unavoidable error in thickness estimation via conventional software.

Our findings imply that segmentation based on DL technique can offer reliable RNFL thickness estimations in both images with and without segmentation error by using U-net network and thickness measurement algorithm. We achieved MAE 2.23 ± 2.1 μm that is less than axial resolution 2.8 μm for OCT conventional algorithms. According to our hypothesis, the proposed method segmented RNFL similarly to ophthalmologists by a DSC score of 0.91. Such a method could prove useful in clinical practice to determine RNFL thickness without the need to refine segmentation, thus avoiding the time-consuming process of segmentation.

### Ethical Considerations

All procedures implemented in studies involving human participants were done in accordance with the ethical standards of the Research Ethics Committees of School of Medicine, Tehran University of Medical Sciences under “IR.TUMS.MEDICINE.REC.1398.827”.

### Financial Support and Sponsorship

None.

### Conflicts of Interest

None.

All authors read and approved the final manuscript.
